# Clinical usefulness of four-dimensional dynamic ventilation CT for borderline resectable locally advanced esophageal cancer

**DOI:** 10.1007/s11604-024-01678-1

**Published:** 2024-10-19

**Authors:** Shioto Oda, Hirofumi Kuno, Takeo Fujita, Takashi Hiyama, Daisuke Kotani, Tomohiro Kadota, Shingo Sakashita, Tatsushi Kobayashi

**Affiliations:** 1 Department of Diagnostic Radiology, National Cancer Center Hospital East, 6-5-1 Kashiwanoha, Kashiwa, Chiba 277-8577 Japan; 2 Department of Esophageal Surgery, National Cancer Center Hospital East, 6-5-1 Kashiwanoha, Kashiwa, Chiba 277-8577 Japan; 3 Department of Gastrointestinal Oncology, National Cancer Center Hospital East, 6-5-1 Kashiwanoha, Kashiwa, Chiba 277-8577 Japan; 4 Department of Gastroenterology and Endoscopy, National Cancer Center Hospital East, 6-5-1 Kashiwanoha, Kashiwa, Chiba 277-8577 Japan; 5 Division of Pathology, Exploratory Oncology Research and Clinical Trial Center, National Cancer Center, 6-5-1 Kashiwanoha, Kashiwa, Chiba 277-8577 Japan

**Keywords:** Esophageal cancer, Four-dimensional dynamic ventilation CT, Oncology, Esophagus

## Abstract

**Purpose:**

This study aimed to evaluate the clinical significance of four-dimensional dynamic ventilation CT (4DCT) for assessing resectability in borderline resectable locally advanced esophageal cancer (BR-LAEC) and confirmed the pathological validity of the 4DCT results in surgery without prior treatment.

**Materials and methods:**

We retrospectively reviewed 128 patients (107 men; median age, 68 [range, 43–89] years) diagnosed with BR-LAEC on initial conventional CT (i-CT). These patients were initially classified into three categories: BR1 (closer to resectable), BR2 (resectability not assessable), or BR3 (closer to unresectable). Subsequent 4DCT reclassified patients as either resectable or unresectable within 1 week of i-CT. We analyzed the diagnostic shift induced by 4DCT. Additionally, 18 patients who underwent surgery without prior treatment were evaluated using 4DCT and pathological outcomes.

**Results:**

4DCT reclassified patients with BR-LAEC as resectable (57.0%; 73/128) and unresectable (43.0%; 55/128). Of 53 patients initially classified as BR1, 32.1% (17/53) were reclassified as unresectable, and of 47 patients initially classified as BR3, 46.8% (22/47) were reclassified as resectable. Among 28 patients initially classified as BR2, 53.6% (15/27) were reclassified as resectable and 46.4% (13/27) as unresectable. In the surgery-only cohort of 18 patients, 9 were initially classified as BR1 and 9 as BR2, and all were reclassified as resectable. These patients were pathologically confirmed to have resectable disease.

**Conclusions:**

4DCT may provide information complementary to that provided by initial conventional CT in assessing resectability among patients with BR-LAEC, and could be a useful adjunct tool for guiding clinical decisions in this patient population.

**Supplementary Information:**

The online version contains supplementary material available at 10.1007/s11604-024-01678-1.

## Introduction

Esophageal cancer was the seventh most prevalent malignancy and seventh leading cause of cancer-related mortality worldwide in 2022 [5]. Despite progress in public health and multimodality treatment, it remains a devastating disease, with a 5 year overall survival rate of 45.0–46.5% [6, 9], partly because 60.1% of esophageal cancer cases are diagnosed at an advanced stage [12].

In the treatment of locally advanced esophageal cancer (LAEC), the pre-treatment assessment of invasion into critical organs is crucial for determining resectability. Based on this assessment, LAEC is classified as either resectable or unresectable, with distinct treatment strategies for each category. For resectable LAEC, standard treatments include neoadjuvant chemotherapy (NAC) with either triplet or doublet chemotherapy, and neoadjuvant chemoradiotherapy (NACRT) regardless of histological type [1–4, 6, 7, 10, 11]; in Japan, NAC is the standard treatment, while in Western countries, NACRT is the standard treatment. For unresectable LAEC, chemoradiotherapy (CRT) or chemotherapy (Cx) is the standard approach. Recent results from the JCOG1109 study have shown that, for squamous cell carcinoma (SCC) of LAEC, triplet NAC improves prognosis more effectively than doublet NAC or NACRT [7]. This suggests that NAC will become increasingly important in future treatment protocols, confirming its role as the standard of care for resectable LAEC.

Given this clinical background, pre-treatment CT assessment to determine resectability is crucial for selecting the appropriate treatment strategy for LAEC. However, it is often challenging to distinguish between resectable and unresectable cases. To address this, the Japan Esophageal Society introduced the concept of “borderline resectable” in the 2022 esophageal cancer practice guidelines for cases that are difficult to classify as either resectable or unresectable [8]. Diagnostic concordance rates among the seven working members of the handling protocol committee were 71.4–100% for cases classified as resectable and unresectable, but only 57.1% for BR-LAECs. Although traditional methods for assessing aortic invasion have been established [15], their diagnostic performance is limited, and standardized methods for evaluating the resectability of other vital organs have not been established.

Four-dimensional dynamic ventilation CT (4DCT) offers a novel approach to imaging and capturing information about anatomy, physiological processes, and motion [16–19]. Currently, 4DCT is used in diagnostic radiology for precise evaluation of joint, cardiac, and pulmonary neoplastic diseases [20–22]. It also plays a role in radiation therapy by enhancing safety during the irradiation of masses in areas susceptible to ventilation motion [16–19, 23, 24]. We hypothesized that 4DCT could serve as a valuable tool for the accurate assessment of LAEC resectability because it is an advanced method that can dynamically capture changes in the relationship between tumor and critical organ caused by ventilation motion.

This study aimed to investigate the efficacy of 4DCT in assessing the resectability of BR-LAEC, assess its influence on therapeutic options, and verify its accuracy by comparing its findings with those of pathological assessments.

## Materials and methods

The institutional ethical review board approved this retrospective study and waived the requirement for informed consent (2020–469). The department received a research grant from Canon Medical Systems (Otawara, Japan). This work was technically supported by Canon Medical Systems. The corresponding author had complete control of the data and the information submitted for publication.

### Study sample

**Fig. 1:**
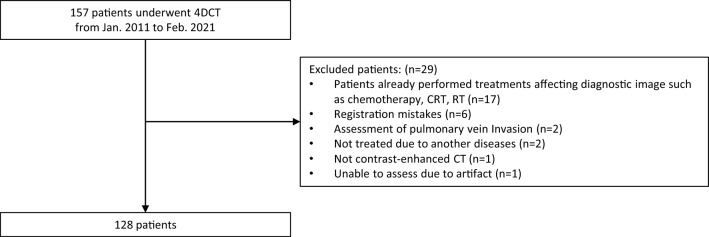
Flowchart of patient selection. *4DCT* four-dimensional dynamic ventilation CT, *CRT* combined chemoradiotherapy, *RT* radiotherapy

This retrospective study included eligible consecutive patients who underwent 4DCT at our institution to evaluate the resectability of BR-LAEC from January 2011 to April 2021. The inclusion criteria were borderline resection on initial conventional CT (i-CT) and consideration for surgery following multidisciplinary discussions among the Departments of Diagnostic Radiology, Endoscopy, Esophageal Surgery, Radiotherapy, and Gastrointestinal Oncology. The 4DCT was performed within a week of this decision. The exclusion criteria are shown in Fig. 1. All patients received NAC, CRT, radiotherapy only (RT), or surgery only (Ope) as initial radical treatment.

The clinical tumor category (clinical TNM category) was determined using CT and endoscopic ultrasonography based on the 7th/8th edition of the Union for International Cancer Control staging system, which has consistent criteria for T and N categories. All patients received NAC, CRT, RT, or Ope as the initial radical treatment. For NAC or CRT, oncologists tailored chemotherapy regimens based on individual patient factors. Adjustments were made in cases of severe hematological toxicity or significant tumor growth. The surgery involved transthoracic esophagectomy with two- or three-field lymphadenectomies. Patients unsuitable for chemotherapy underwent Ope or RT. The treatment decisions were maximally aligned with patient preferences.

### CT image acquisition

#### Initial conventional CT technique

All patients underwent preoperative i-CT scans using area-detector CT scanners (Aquilion ONE Vision; Canon Medical Systems, Otawara, Japan) with the following scanning parameters: tube voltage, 120 kV; gantry rotation time, 0.5 s; detector collimation, 0.5 mm; and helical pitch (pitch factor), 65.0 (0.813). Automatic exposure control was applied to adjust the tube current and maintain a user-specified noise level in the imaging data; the target noise value (expressed as standard deviation) was set to 11. Additionally, patients were intravenously administered 60–150 mL (600 mg I/kg) of iodinated contrast medium (iohexol 300; Ioverin, Teva Takeda Pharma or iopamidol 370; Oypalomin, Konica Minolta) at a rate of 2.5 mL/s and were scanned at the portal phase (70 s after injection). Axial CT images were reconstructed using 5-mm slice thickness and 512 × 512 matrices, and the field of view was adjusted according to the body size of the patients (minimum, 300 mm; maximum, 420 mm).

#### Four-dimensional CT (4DCT) techniques

4DCT imaging was performed using 320 area-detector CT scanners (Aquilion One; Canon Medical Systems until November 1, 2019, and thereafter using Aquilion One Vision; Canon Medical Systems). The scanning parameters were as follows: tube voltage, 120 kV; tube current, 80 mA with a rotation time of 0.35 s (29 mAs). Images were captured in 11 phases, covering a 16 cm range from the cranial to the caudal aspect of the target area. The capture lasted 3 s, spanning from the patient’s maximum exhalation to maximum inhalation while intentionally performing chest breathing, and took place between 60 and 180 s after contrast injection. The radiation exposure was recorded as the CT dose index volume of 89.30 mGy and a dose length product of 1428.20 mGy·cm.

In our institution, we determined that the clinical benefits of 4DCT for BR-LAEC outweigh the disadvantages of radiation exposure, and we conducted 4DCT after obtaining informed consent from the patients. The 4DCT imaging protocol was based on a previous study (*n* = 15), wherein patient consent was obtained which was approved by the institutional review board (IRB no. 21–017).

### Imaging evaluation

#### Imaging evaluation of initial CT

Patients with BR-LAEC were classified into three categories determined by the tumor board on i-CT: BR1; closer to resectable, BR2; resectability not assessable, and BR3; closer to unresectable. The most severe classification was selected if multiple critical organs showed borderline resectability. The main targets of the resectability assessment were aortic and tracheal/bronchial invasion. Aortic invasion was evaluated based on the Picus angle [15], which is the contact angle between the tumor and aorta, contact length, and boundary unclearness of the contact area between the tumor and the aortic wall. Tracheal and bronchial invasions were evaluated based on the compression and enclosure caused by the tumor and contact length. The detailed evaluation criteria are described below.

##### Aortic invasion

Supplementary Fig. 1 provides an overview of aortic invasion. All BR1-3 cases were characterized by an unclear contact boundary with wide contact. BR1 was diagnosed when the contact angle was < 90° with a contact length of ≥ 10 mm, or when the contact angle was 90°–110° with a contact length of ≤ 5 mm. BR2 was diagnosed when the contact angle was 90°–110° with a contact length of 5–10 mm, or when the contact angle exceeded 110° with a contact length of ≤ 5 mm. BR3 was diagnosed when the contact angle was 90°–110° with a contact length of 10–20 mm, or when the contact angle exceeded 110° with a contact length of 5–10 mm. Cases were classified as unresectable when the contact angle was 90°–110° with a contact length of ≥ 20 mm, or when the contact angle exceeded 110° with a contact length of > 10 mm.

##### Tracheal and bronchial invasion

Supplementary Fig. 2 provides an overview of tracheal and bronchial invasion. BR3 was diagnosed when the stratification of the contact boundary with the airway by the primary lesion was unclear, and the airway was significantly compressed, displaced, and narrowed. BR1 was diagnosed when there was a wide contact length with significant compression but without encirclement of the trachea/bronchus or there was a wide contact length with encirclement but no compression. BR2 was diagnosed when the tumor exhibited characteristics of either BR1 or BR3.

#### Imaging evaluation of 4DCT

In this study, we used software (Vitrea workstation version 7.14.3.197; Canon Medical Systems, Otawara, Japan) to analyze cross-sectional and three-dimensional (3D) image changes associated with respiratory motion. Two radiologists with 12 (*S.O.*) and 29 (*T.Kobayashi*) years of experience in oncological diagnostic radiology conducted the evaluation. The main targets were aortic and tracheal/bronchial infiltrations, which were classified as resectable and unresectable, respectively, after discussion. Aortic invasion was assessed based on whether the enclosure of the aorta changed with the respiratory motion. Tracheal/bronchial invasion was assessed based on whether enclosure and compression changed with respiratory motion. The detailed evaluation criteria are described below and in Supplementary Fig. 3. A bar chart was used to depict the correlation among i-CT, 4DCT, and treatment outcomes.

##### Aortic invasion

First, the appropriate sections were determined. The short-axis image that best displays the contact area between the aorta and tumor was identified, and the slice with a significant contact area and encircling appearance on the axial images was selected. Second, if the tumor encirclement of the aorta changed with respiration, both on the axial slice from the first step and on the sagittal image, non-infiltration was determined.

##### Tracheal/bronchial invasion

First, the appropriate sections were determined. The short-axis image that best displays the contact area between the trachea/bronchus and tumor was identified, and the slice with a significant contact area and encircling appearance on the axial images were selected. Second, there was a transition to the coronal image from the axial slice in the first step, and the image was adjusted to make the bronchus horizontal. Using the resulting oblique sagittal images, if the compression of the tumor on the bronchial section was alleviated by respiratory movement or if the tumor and bronchus moved independently owing to respiration, non-infiltration was determined.

### Histopathological evaluation

Two experienced pathologists (each with > 10 years of experience in oncological pathology) routinely diagnosed the surgically resected specimens following the Union for International Cancer Control 7th edition guidelines. When the evaluation of surrounding organ involvement was difficult on pathology, pT was determined based on intraoperative findings. Patients with pT3 showed invasion of the esophageal adventitia but not the adjacent organs. Patients with pT4a had pericardial, pulmonary, or pleural invasion, whereas those with pT4b had an invasion of the aorta, trachea, bronchus, or major vessels.

**Fig. 2:**
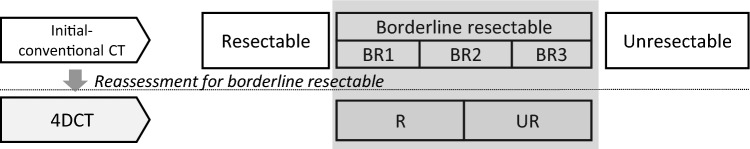
Relationship between radiological resectability evaluation in this study on initial conventional CT and four-dimensional dynamic ventilation CT (4DCT). In cases where there is clear infiltration of vital organs, such as major vessel infiltration or tracheal infiltration, it is classified as “unresectable” (UR). If there is a clear absence of such infiltration, it is classified as “resectable” (R). However, if it is difficult to determine, it is classified as “borderline resectable” (BR). The “BR” category includes cases where vital organ infiltration is suspected. In this study, BR-LAECs were further classified as BR1 (closer to resectable), BR2 (resectability not assessable), or BR3 (closer to unresectable) on initial conventional CT. Subsequently, the resectability on 4DCT was reassessed and diagnosed

The correlation between 4DCT findings and pathological outcomes for evaluating resectability was examined in a surgery-only subgroup. Surgery was performed within a median of 17 (range, 3–39) days after the decision was made. Figure 2 and Supplementary Fig. 4 show the relationship between the radiological resectability of i-CT/4DCT and tumor-node-metastasis (TNM) classification.

### Statistical analyses

The Mann–Whitney U and chi-squared tests were used to compare demographic and clinical characteristics for continuous and categorical variables, respectively. For inter-rater agreement of the resectability assessment on 4DCT, 60 patients with suspected aortic (30 patients) and tracheal/bronchial invasion (30 patients) were selected. Two board-certified radiologists (with 12 [S.O.] and 18 [T.H.] years of experience in oncological diagnostic radiology) independently assessed each case. The ratings were as follows: (1), suspected unresectable, (2), likely unresectable but not certain; (3), likely resectable but not certain; and (4), suspected resectable. Inter-rater agreement was quantified using the weighted kappa statistic and a quadratic weighting scheme. The weighted kappa interpretation followed the criteria of Landis et al. [25]: 0.81–1.00, almost perfect; 0.61–0.80, substantial; 0.41–0.60, moderate; 0.21–0.40, fair; and 0.00–0.20, slight.

Statistical analyses were performed using EZR, which is a graphical interface for R [26]. Statistical significance was set at *p* ≤ 0.05.

## Results

### Study sample

Initially, we identified 157 patients; 29 were excluded for the following reasons: prior treatments, such as chemotherapy or radiation, that might affect imaging or pathological evaluation (*n* = 17), registration errors (*n* = 6), subsequent discovery of other diseases or metastases (*n* = 2), assessment for non-vital organ (pulmonary vein) involvement (*n* = 2), absence of contrast enhancement (*n* = 1), and unassessability for significant artifacts (*n* = 1) (Fig. 1). Finally, 128 patients (107 men and 21 women; mean age, 68 [range, 43–89] years) were included.

### Patient characteristics

The patient characteristics are shown in Table 1. The median tumor size was 60 (range, 20–115) mm. Based on i-CT, 53 (41.4%) were classified as BR1, 28 (21.9%) as BR2, and 47 (36.7%) as BR3. The 4DCT evaluation categorized 73 (57.0%) tumors as resectable and 55 (43.0%) as unresectable. Treatment options included NAC (54 [42.2%]), CRT (53 [41.4%]), Ope (18 [14.1%]), and RT (3 [2.3%]). The mortality rate during follow-up was 56 (43.8%), with a median survival period of 17 (range, 0–103) months. Significant differences in age, 4DCT category, and overall survival were observed across treatment subgroups.

**Table 1:** Patient characteristics in the study cohort

Variables	Total *n* (%)	Treatment				*p*-value
		NAC	CRT	Ope	RT	
Total patients	128	54	53	18	3	
Sex (%)						
F	21 (16.3)	12 (22.2)	5 (9.4)	3 (16.7)	1 (33.3)	0.279
M	107 (83.6)	42 (77.8)	48 (90.6)	15 (83.3)	2 (66.7)	
Age (year)^†^	68 [43–89]	66 [47–79]	67 [43–83]	74 [60–84]	82 [66–89]	0.009^*^
Size (mm)^†^	60 [20–115]	60 [30–110]	65 [30–115]	52.50 [20–100]	70 [30–90]	0.163
i-CT						
BR1	53 (41.4)	24 (44.4)	17 (32.1)	9 (50.0)	3 (100.0)	0.057
BR2	28 (21.9)	13 (24.1)	15 (28.3)	0 (0.0)	0 (0.0)	
BR3	47 (36.7)	17 (31.5)	21 (39.6)	9 (50.0)	0 (0.0)	
cN						
0	5 (3.9)	3 (5.6)	0 (0.0)	2 (11.1)	0 (0.0)	0.099
1	38 (29.7)	10 (18.5)	19 (35.8)	9 (50.0)	0 (0.0)	
2	56 (43.8)	27 (50.0)	22 (41.5)	5 (27.8)	2 (66.7)	
3	29 (22.7)	14 (25.9)	12 (22.6)	2 (11.1)	1 (33.3)	
Location						
Cervical	6 (4.7)	1 (1.9)	4 (7.5)	0 (0.0)	1 (33.3)	0.087
Upper	51 (39.8)	17 (31.5)	24 (45.3)	9 (50.0)	1 (33.3)	
Middle	68 (53.1)	33 (61.1)	25 (47.2)	9 (50.0)	1 (33.3)	
Lower	3 (2.3)	3 (5.6)	0 (0.0)	0 (0.0)	0 (0.0)	
4DCT						
R	73 (57.0)	47 (87.0)	8 (15.1)	18 (100.0)	0 (0.0)	< 0.001^*^
UR	55 (43.0)	7 (13.0)	45 (84.9)	0 (0.0)	3 (100.0)	
OS event						
Alive	72 (56.2)	36 (66.7)	28 (52.8)	6 (33.3)	2 (66.7)	0.086
Death	56 (43.8)	18 (33.3)	25 (47.2)	12 (66.7)	1 (33.3)	
OS period (months)^†^	17 [0–103]	19 [0–101]	17 [0–103]	16 [2–101]	2 [2, 3]	0.039^*^

Note. Unless otherwise indicated, the data represent the number of patients

The Mann–Whitney *U* test was used for continuous data, and categorical data were compared using the Chi-Squared test

*i-CT* initial conventional CT, *BR1* borderline resectable, closer to resectable, *BR2* resectability not assessable, *BR3* closer to unresectable, *cN* clinical N factor, cervical, cervical esophagus, *Upper* upper thoracic esophagus, *Middle* middle thoracic esophagus, *Lower* lower thoracic esophagus, *4DCT* four-dimensional CT, *R* resectable, *UR* unresectable, *NAC* neoadjuvant chemotherapy, *CRT* combined chemoradiotherapy, *Ope* surgery only, *RT* radiotherapy, *OS* overall survival

†Data are presented as medians and ranges

*Statistically significant results

### Diagnostic and therapeutic outcomes: comparing conventional CT and 4DCT

**Fig. 3:**
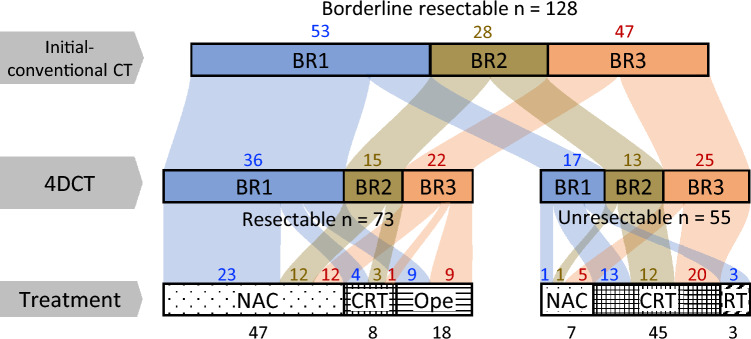
Bar chart of the correlation between initial conventional CT and four-dimensional ventilation CT (4DCT) and treatment. The treatment decision was made by integrating 4DCT results with clinical judgment. The number in the middle refers to the number of patients. *BR1* borderline resectable, closer to resectable, *BR2* resectability not assessable, *BR3* closer to unresectable, *R* resectable, *UR* unresectable

Figure 3 shows a bar chart of the correlation between i-CT and 4DCT and treatment. The 4DCT changed evaluations in 52.3% of the patients; 32.1% of the patients classified as having BR1 on i-CT were upgraded to unresectable on 4DCT, and 46.8% of the patients classified as having BR3 were downgraded to resectable. Patients classified as having BR2 accounted for 21.9% of all patients, and 4DCT was divided into resectable (53.6%) and unresectable (46.4%). For patients deemed resectable on 4DCT, the treatment options were NAC (64.4%), CRT (11.0%), and OPE (24.7%). For patients with tumors deemed unresectable on 4DCT, the distributions were NAC (12.7%), CRT (81.8%), and RT (5.5%). Of the 18 patients in the Ope subgroup, half were originally classified as having BR3.

**Fig. 4:**
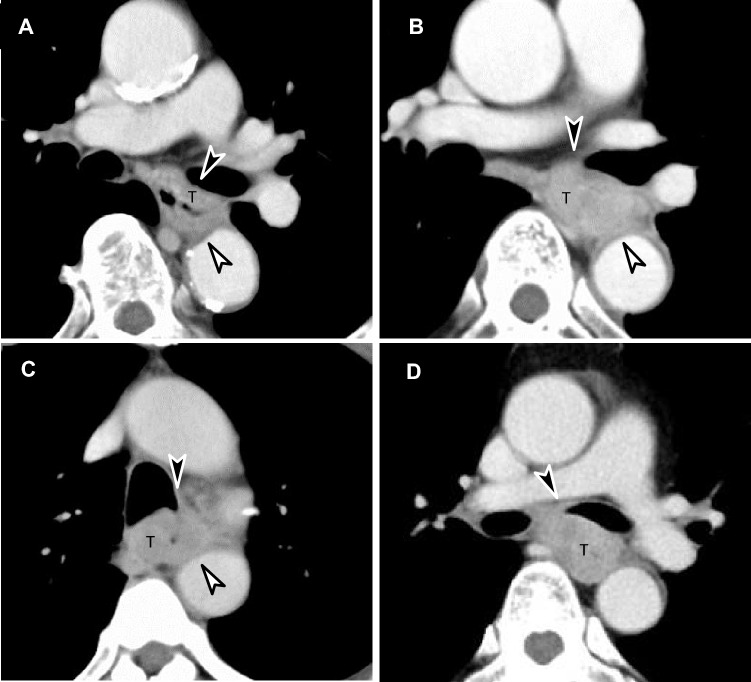
Images of borderline resectable locally advanced esophageal cancer (BR-LAEC) on initial conventional CT (i-CT). Axial contrast-enhanced portal venous phase CT images at the level of the thoracic esophagus (**a**–**d**). The black arrowhead represents the suspected site of the main bronchial invasion, and the white arrowhead represents the suspected site of aortic invasion. **a** BR-LAEC with 100 mm of craniocaudal length in a 77-year-old man, with the tumor slightly surrounding the bronchus without compression (Br-BR1) and surrounding the aorta, but not widely (Ao-BR1). **b** BR-LAEC with 45 mm of craniocaudal length in a 60-year-old man, with the tumor slightly surrounding the bronchus with compression (BR-BR2) and surrounding the aorta widely (Ao-BR2). **c** BR-LAEC with 60 mm of craniocaudal length in a 63-year-old man, with a tumor highly surrounding the bronchus (Br-BR3) and surrounding the aorta widely (Ao-BR2). **d** BR-LAEC with 40 mm of craniocaudal length in a 56-year-old man, with the tumor highly surrounding the bronchus (Br-BR3). The result of 4DCT is that the first three cases are all resectable (**a**, **b**, **c**), and the last case is unresectable (**d**)

**Fig. 5:**
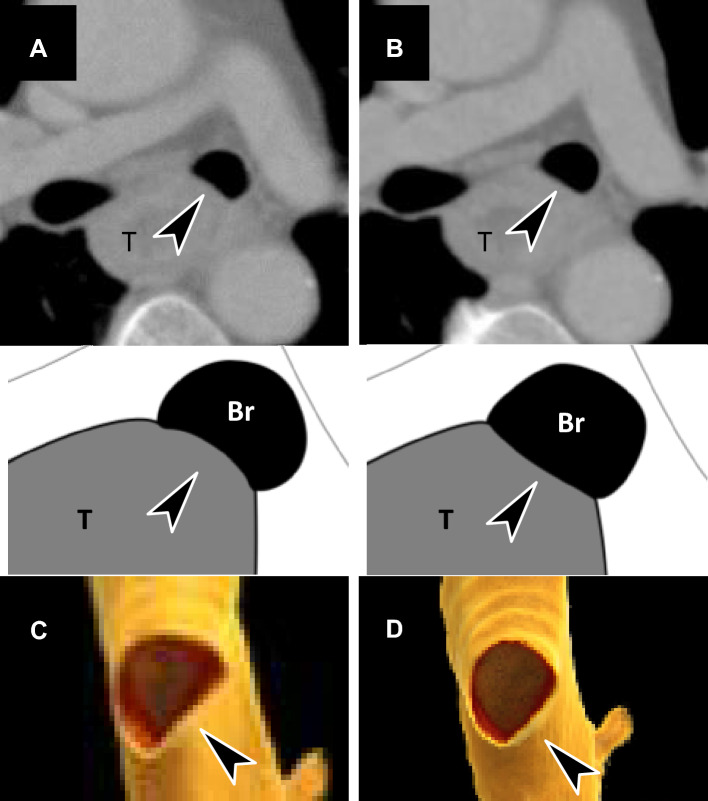
Axial and volume rendering images of a resectable locally advanced esophageal carcinoma (LAEC) on four-dimensional ventilation CT (4DCT) in a 63-year-old man (**a**–**d**). This is a case of suspected left bronchial invasion (arrowhead) examined by 4DCT; bronchial membranous part is compressed by the tumor in the maximum expiratory phase (**a**, **c**), and the finding is released in the maximum inspiratory phase (**b**, **d**), suggesting resectability for bronchial invasion. Supplemental Material 1 corresponds to this movie. *T* tumor, *Br* bronchus

**Fig. 6:**
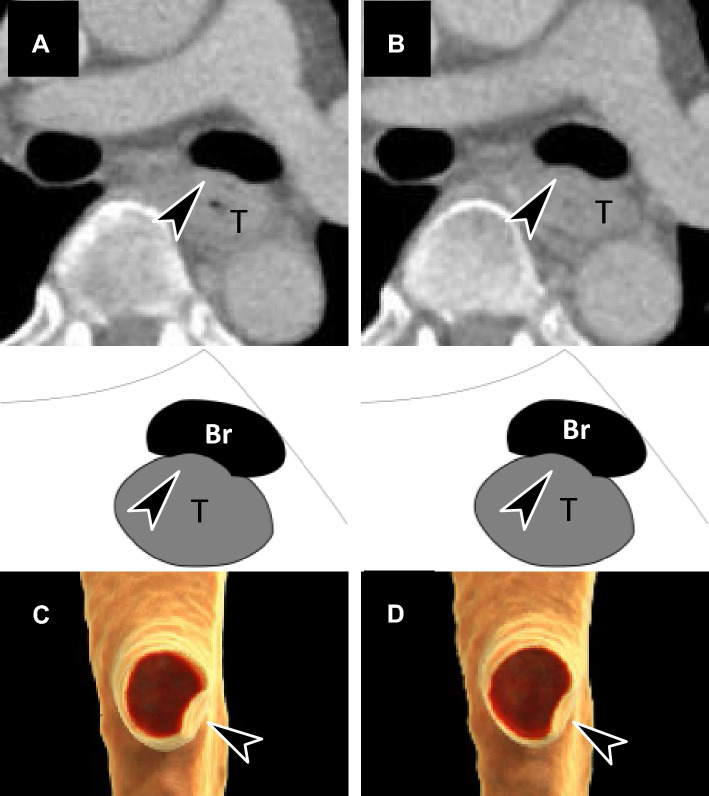
Axial and volume rendering images of a resectable locally advanced esophageal carcinoma (LAEC) on four-dimensional ventilation CT (4DCT) in a 49-year-old man (**a**–**d**). This is a case of suspected left bronchial invasion (arrowhead) examined by 4DCT; bronchial membranous part is compressed by the tumor in the maximum expiratory phase (**a**, **c**), and the finding is not released in the maximum inspiratory phase (**b**, **d**), suggesting unresectability for bronchial invasion. Supplemental Material 2 corresponds to this movie. *T* tumor, *Br* bronchus

Figure 4 shows the images of the aortic and tracheal/bronchial BR1, BR2, and BR3 cases from i-CT with 4DCT outcomes. Figure 5 shows a resectable case on 4DCT, and Supplementary Material 1 provides a 3D rendering video of movement during respiration. The patient, in this case, was initially evaluated as having BR3 on i-CT and underwent surgery without preoperative chemotherapy according to the 4DCT evaluation, which proved to be pathologically resectable. Figure 6 shows unresectable cases on 4DCT, and Supplementary Material 2 provides a 3D rendering video of movement during respiration. The patient in this case was initially evaluated as having BR1 on i-CT and received induction NAC and underwent surgery after a confirmed response to the treatment. There was no invasion of the vital organs.

### Diagnostic and pathological outcomes: comparing 4DCT and pathological assessment

Table 2 shows the patient characteristics in the surgery-only subgroup. All 18 tumors were deemed resectable on 4DCT. Pathological evaluation revealed the following: pT2, 5.6%; pT3, 83.3%; and pT4a, 11.1%. None of the patients had pT4b disease. Of the two patients with pT4a, one exhibited pericardial infiltration and the other exhibited pulmonary infiltration.

**Table 2:** Patient characteristics of the surgery-only subgroup

Variable	Overall
Total patients	18 (100.0)
Sex	
F	3 (16.7)
M	15 (83.3)
Age (years)^†^	74 [60–84]
Size (mm)^†^	52.5 [20–100]
i-CT	
BR1	9 (50.0)
BR2	0 (0.0)
BR3	9 (50.0)
cN	
0	2 (11.1)
1	9 (50.0)
2	5 (27.8)
3	2 (11.1)
Location	
Cervical	0 (0.0)
Upper	9 (50.0)
Middle	9 (50.0)
Lower	0 (0.0)
pT	
2	1 (5.6)
3	15 (83.3)
4a	2 (11.1)
4b	0 (0.0)
OS event	
Alive	6 (33.3)
Death	12 (66.7)
OS period (months)^†^	16 [2–101]

Note. Unless otherwise indicated, the data represent the number of patients

*i-CT* initial conventional CT, *BR1* borderline resectable, closer to resectable, *BR2* resectability not assessable, *BR3* closer to unresectable, *cN* clinical N factor, *pT* pathological T factor, Cervical cervical esophagus, *Upper* upper thoracic esophagus, *Middle* middle thoracic esophagus, *Lower* lower thoracic esophagus

†Data are presented as medians and ranges

Supplementary Table S1 shows the clinical characteristics of the NAC and Ope subgroups based on the pathological evaluation. The two patients with pT4b, both belonging to the NAC group, were deemed to have resectable tumors on 4DCT: one had macroscopic tracheal invasion at the time of surgery, and the other had microscopic tracheal invasion on pathological evaluation after resection.

### Inter-rater agreement

The results of the inter-rater agreement of the resectability assessment on 4DCT were as follows: For suspected aortic invasion, the weighted kappa with quadratic weights was 0.7482 (95% confidence interval [CI], 0.5929 to 0.9035). For suspected tracheal/bronchial invasion, the weighted kappa was 0.6816 (95% CI, 0.5149 to 0.8483), indicating substantial agreement in both patients.

## Discussion

Esophageal cancer frequently presents at an advanced stage; thus, determining resectability is crucial for treatment strategy. However, a gold standard for assessing resectability has not yet been established. To address this, a classification of borderline resectability has recently been introduced for clinical treatment in the 2022 Esophageal Cancer Practice Guidelines [8]. In this study, we focused on the changes between the tumor and surrounding structures with respiratory motion and adopted 4DCT to evaluate its clinical performance in assessing resectability. Our findings revealed significant shifts in resectability assessments for more than half of the patients; approximately one-third of the patients initially classified as closer to resectable were later reclassified as unresectable on 4DCT, nearly half of those initially classified as closer to unresectable were later reclassified as resectable, and those initially classified as resectability not assessable were later divided equally between resectable and unresectable. Subsequent pathological analysis confirmed that all tumors reclassified as resectable on 4DCT were pathologically resectable. These findings indicate that 4DCT assessment of resectability may be reliable and has the potential to help make more appropriate treatment decisions for BR-LAEC.

The pretreatment assessment of resectability in LAEC using CT imaging has focused on evaluating vital organs [27, 28], particularly the aorta [8] and trachea/bronchus invasion [8]. While the method of Picus et al. [15] for assessing aortic invasion has become standard, the assessment of tracheal/bronchial invasion remains particularly challenging. Despite these efforts, there is no consensus on an accurate pre-treatment resectability assessment method, making appropriate treatment selection difficult. In response to this need, the Japan Esophageal Society introduced the concept of “borderline resectable” for cases that are difficult to classify as either resectable or unresectable [8] (Supplementary Fig. 1). However, the diagnostic concordance rates among the seven working members of the handling protocol committee were significantly lower for BR-LAECs compared to those for resectable and unresectable cases. 4DCT is an advanced method that, in addition to conventional CT evaluation, can assess changes in tumors and critical organs due to respiratory motion. As shown in the provided images and videos, the trachea and bronchi undergo significant morphological changes during different respiratory phases, making it relatively easier to assess the impact of tumors. This technique is expected to improve the accuracy of bronchial invasion assessment and to enhance the overall capability to evaluate surgical resectability comprehensively.

Studies focusing on the resectability of LAEC using modalities other than CT, such as EUS, MRI, and PET, are predominantly conducted in the context of post-treatment preoperative evaluation [31–37]. EUS, MRI, and CT studies by Guo et al. [38] and the MRI study by Haefliger et al. [39] were related to the T-category staging of LAEC before treatment, but they did not specifically evaluate resectability with an emphasis on the invasion of vital organs. EUS is primarily used to evaluate whether early-stage cancer can be treated endoscopically. However, its performance significantly declines when evaluating deeper lesions involving surrounding organs, because the ultrasound waves cannot penetrate these areas effectively. While MRI and PET have been studied for post-treatment preoperative evaluation [35–37], there is a lack of research on their use for pre-treatment assessment. Future studies are anticipated to explore the potential of MRI and PET in this context, given their promising capabilities in imaging and staging.

This study has some limitations. First, the effects of 4DCT assessment on prognosis and the evaluation of its concordance with pathological findings in terms of sensitivity and specificity remain unclear. This uncertainty arises from the design of our study, which, for ethical reasons, lacked a control group that did not undergo 4DCT during the same period. 4DCT is minimally invasive and allows for the same evaluation methods as conventional CT, making it ethically problematic to establish a control group. Additionally, the number of cases without preoperative treatment, where a close correlation between 4DCT assessment and pathological findings could be expected, was limited to 18 cases. This limitation stems from the current standard treatment for LAEC, where upfront resection without preoperative treatment is uncommon. Cases deemed unresectable based on imaging are typically excluded from immediate surgical intervention. To address this issue and gather more conclusive evidence, a large-scale, multicenter collaborative study is necessary. Second, the 4DCT evaluation in this study is subjective. Although the inter-rater reliability was not poor, the inter-rater assessment was not conducted during the initial evaluation owing to the study design. Future studies should consider the development of quantitative evaluation criteria to increase objectivity. Third, the ability to evaluate 4DCT may vary depending on the site of the esophagus. Although patients were asked to intentionally perform thoracic breathing during imaging to maximize respiratory motion in the thoracic area, the thoracic esophagus exhibited more movement than the cervical esophagus. Further research is needed to evaluate this aspect. However, the respiration-induced motions of the tracheal and bronchial membranous portions were sufficient, allowing for the accurate assessment of suspected membranous invasion even in the cervical esophagus region. Fourth, 4DCT has a disadvantage with respect to exposure. This study was conducted with the understanding that the assessment of resectability is clinically necessary. Future studies should consider further limiting eligible cases by incorporating prior imaging studies such as MRI, exploring imaging methods that minimize radiation exposure, or investigating the feasibility of using two-phase imaging at maximum inspiration and expiration as a substitute. If the issue of radiation exposure can be resolved, the inclusion of cases initially deemed unresectable could be considered.

In conclusion, our study demonstrated that the implementation of 4DCT in LAEC altered the preoperative resectability assessment in half of the patients. In the pathological evaluation, all cases in which 4DCT assessed resectability had no macroscopic invasion of the surrounding vital organs, suggesting the potential for a more accurate resectability evaluation.

## Supplementary Information

Below is the link to the electronic supplementary material.

Supplementary file1 (MP4 600 KB) (Movie for Figure 5, Movie1.mp4) Four-dimensional CT of a resectable case in a 63-year-old man with an initial conventional CT suggesting BR3. Movie showing the axial (a), coronal (b), sagittal (c), and three-dimensional rendered (d) images. Static images of the maximum expiratory (e) and inspiratory (f) phases. The compression of the tracheal membranous portion (arrows e, f) gradually resolves during inspiration

Supplementary file2 (MP4 872 KB) (Movie for Figure 6, Movie2.mp4) Four-dimensional CT of an unresectable case in a 49-year-old man, with an initial conventional CT suggesting BR1. Movie showing the axial (a), coronal (b), sagittal (c), and three-dimensional rendered (d) images. Static images of the maximum expiratory (e) and inspiratory (f) phases. The compression of the tracheal membranous portion (arrows e, f) does not resolve during expiration

Supplementary file3 (PNG 137 KB) Imaging evaluation of aortic invasion on initial CT in this study

Supplementary file4 (PNG 178 KB) Imaging evaluation of tracheal and bronchial invasion on initial CT in this study

Supplementary file5 (PNG 253 KB) Imaging evaluation of aortic invasion and Tracheal/Bronchial invasion on 4DCT in this study

Supplementary file6 (PNG 164 KB) Difference in the T factor category between the 12th edition Japanese classification of esophageal cancer and the 8th edition AJCC/UICC TNM classification of Malignant tumors. In 2022, the Japanese classification was revised to its 12th edition, introducing changes to the T factor category evaluation, which had previously been aligned with the AJCC/UICC system. The classification now emphasizes the importance of determining whether surgical resection is feasible in clinical assessment. Consequently, T3 has been subdivided into T3r and T3br, introducing the concept of “borderline resectable”. While this concept exists internationally, it has not yet been integrated into standard treatment protocols. AJCC; American Joint Committee on Cancer, UICC; Union for International Cancer Control

Patient characteristics in the surgery performed subgroup (NAC and Ope)
