# Supplementary figures and images for: Clinical usefulness of four-dimensional dynamic ventilation CT for borderline resectable locally advanced esophageal cancer
Source: Jpn J Radiol. 2024 Oct 19;43(3):434–44. doi: 10.1007/s11604-024-01678-1 (PMC11868203; doi:10.1007/s11604-024-01678-1)

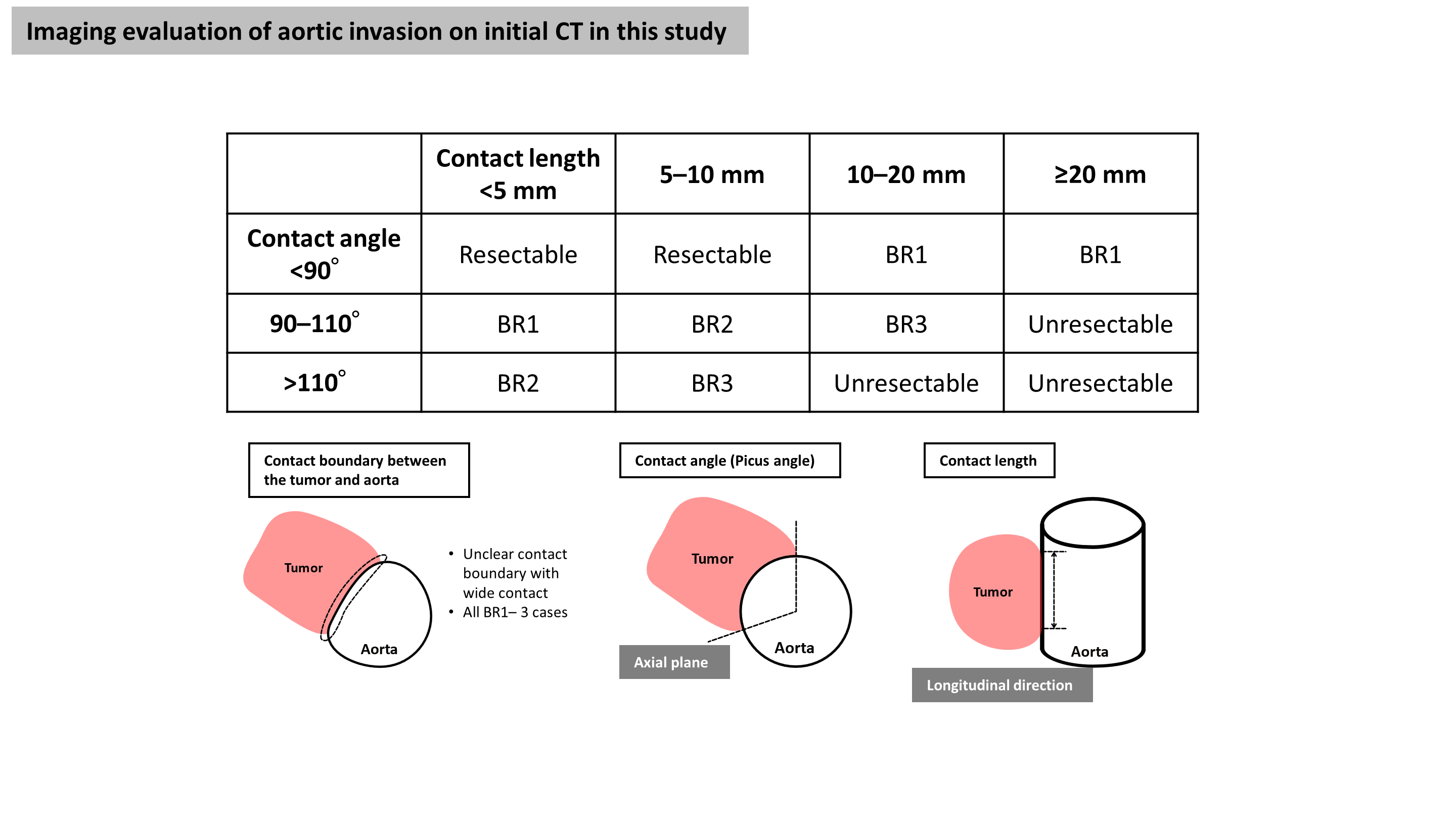

Supplement: Supplementary file 3 — Supplementary file3 (PNG 137 KB) Imaging evaluation of aortic invasion on initial CT in this study [file 11604_2024_1678_MOESM3_ESM.png]

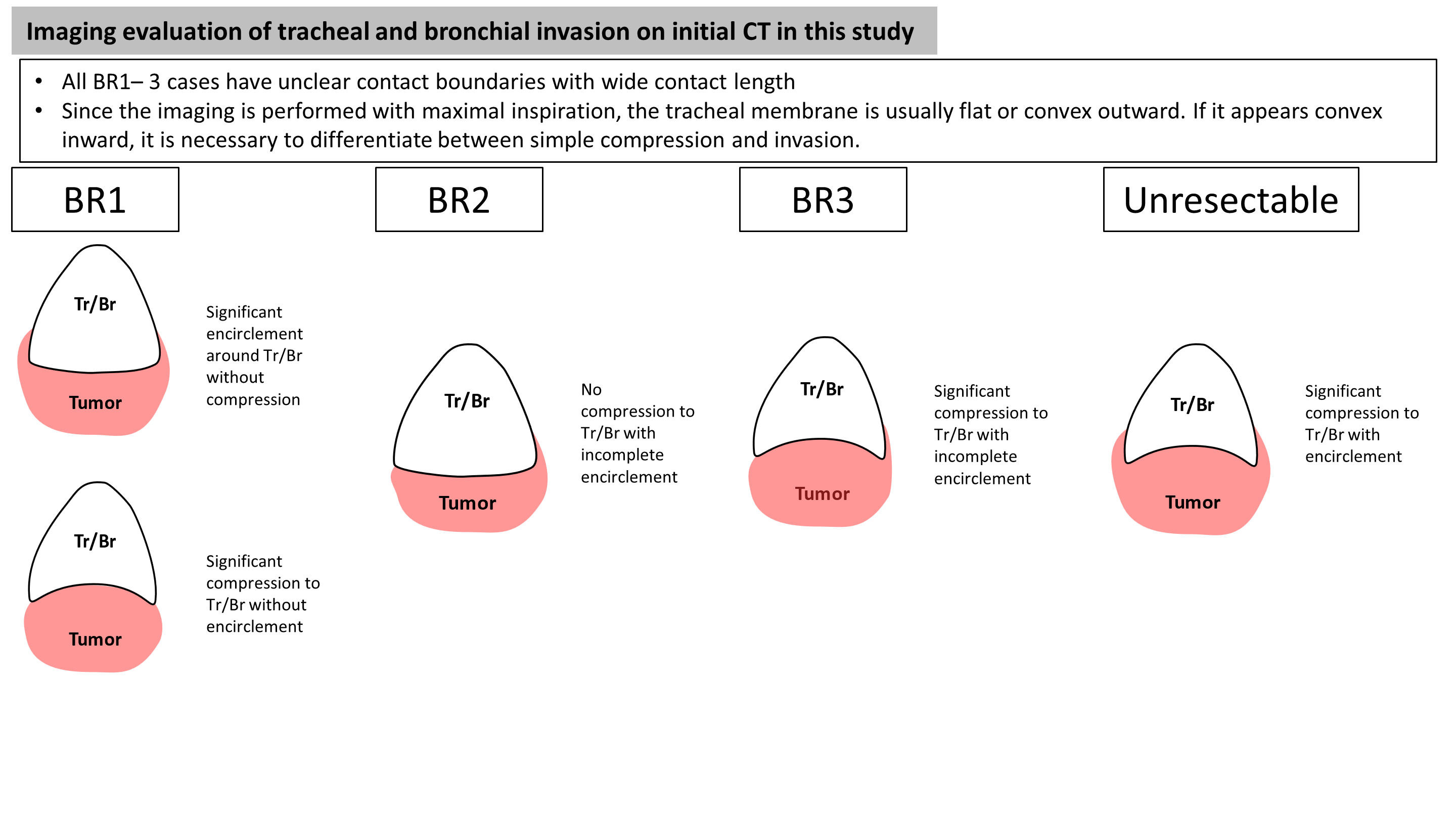

Supplement: Supplementary file 4 — Supplementary file4 (PNG 178 KB) Imaging evaluation of tracheal and bronchial invasion on initial CT in this study [file 11604_2024_1678_MOESM4_ESM.png]

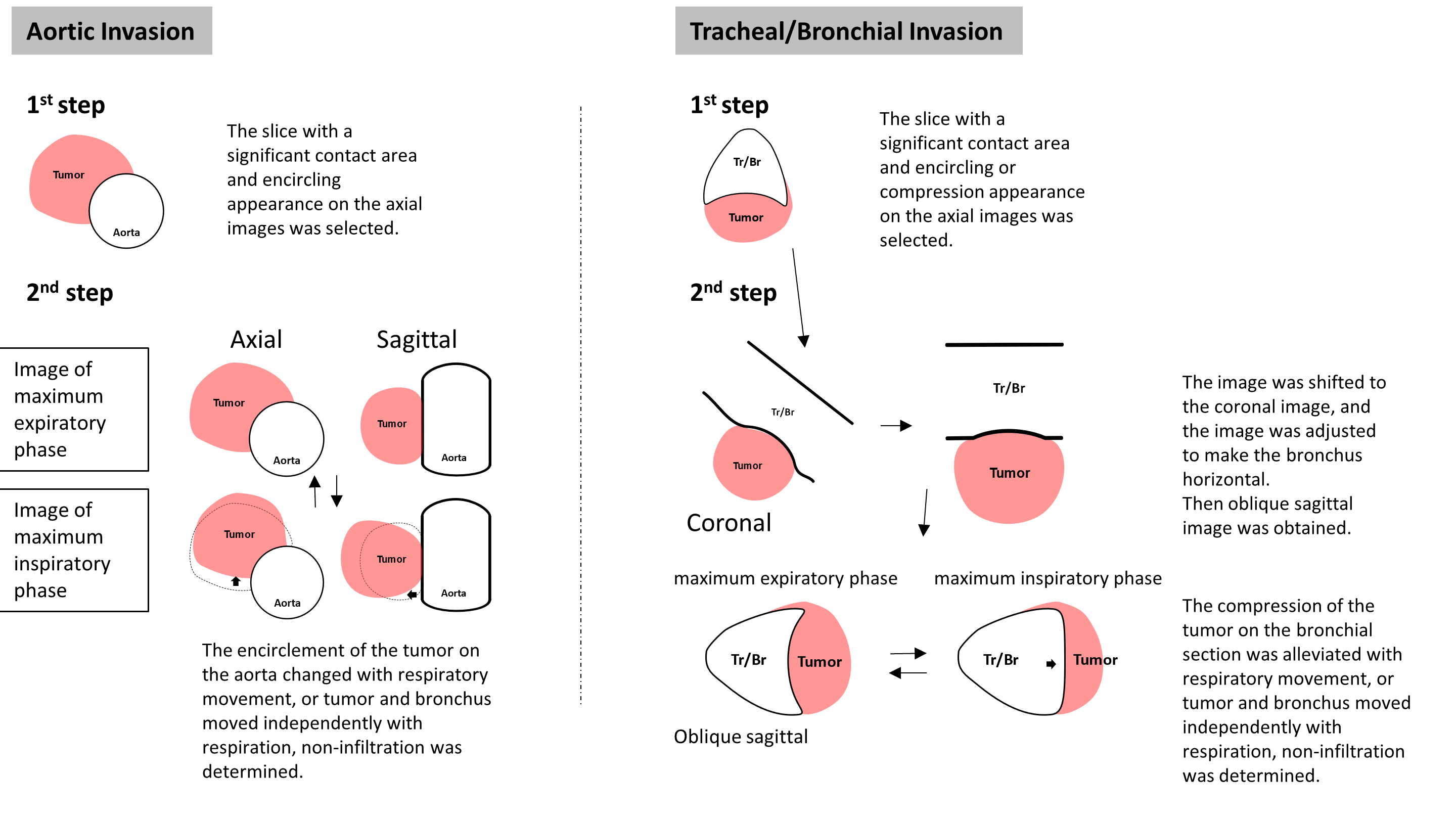

Supplement: Supplementary file 5 — Supplementary file5 (PNG 253 KB) Imaging evaluation of aortic invasion and Tracheal/Bronchial invasion on 4DCT in this study [file 11604_2024_1678_MOESM5_ESM.png]

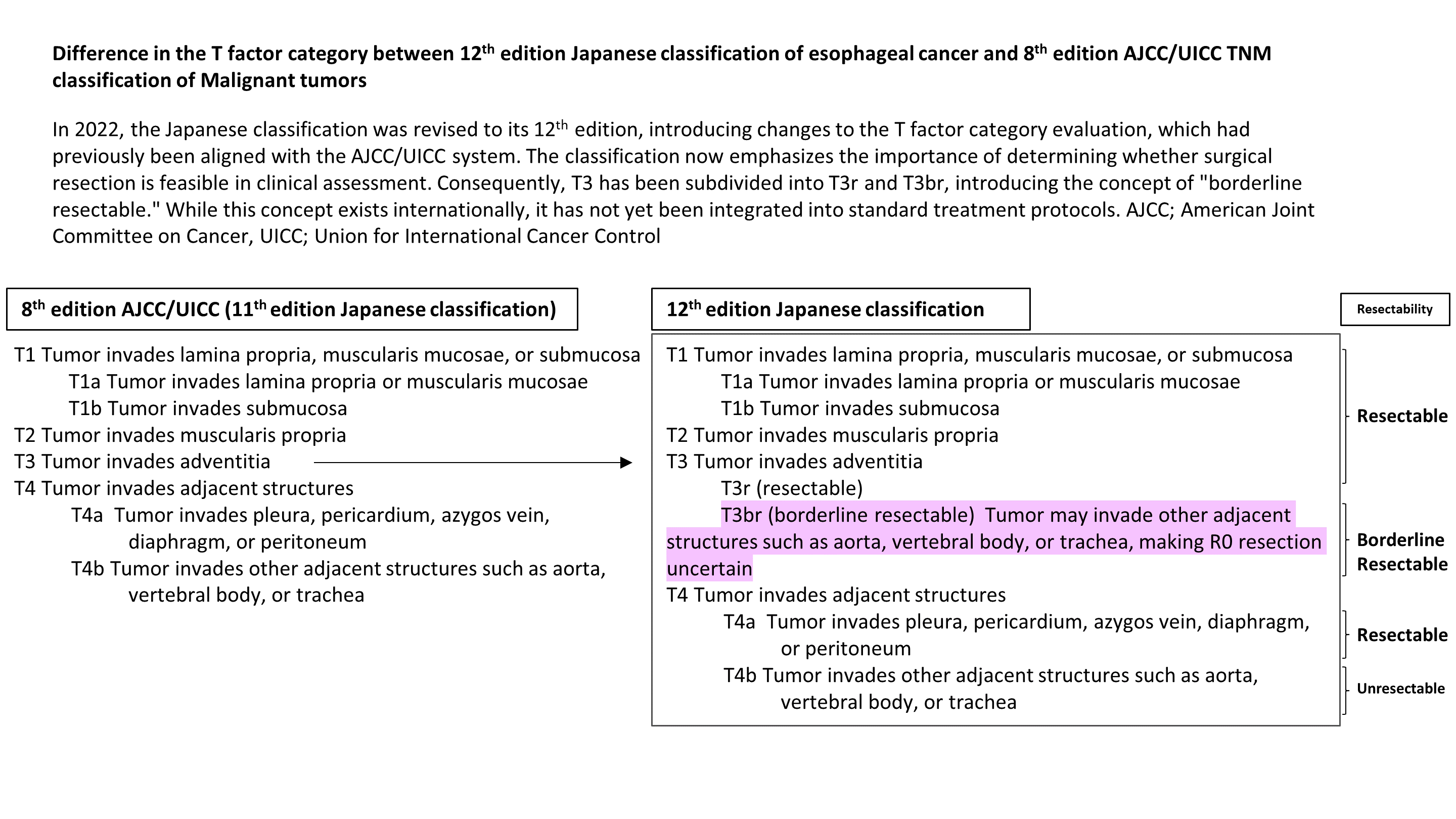

Supplement: Supplementary file 6 — Supplementary file6 (PNG 164 KB) Difference in the T factor category between the 12th edition Japanese classification of esophageal cancer and the 8th edition AJCC/UICC TNM classification of Malignant tumors. In 2022, the Japanese classification was revised to its 12th edition, introducing changes to the T factor category evaluation, which had previously been aligned with the AJCC/UICC system. The classification now emphasizes the importance of determining whether surgical resection is feasible in clinical assessment. Consequently, T3 has been subdivided into T3r and T3br, introducing the concept of “borderline resectable”. While this concept exists internationally, it has not yet been integrated into standard treatment protocols. AJCC; American Joint Committee on Cancer, UICC; Union for International Cancer Control [file 11604_2024_1678_MOESM6_ESM.png]
