# Supplementary material for: Clinical usefulness of four-dimensional dynamic ventilation CT for borderline resectable locally advanced esophageal cancer
Source: Jpn J Radiol. 2024 Oct 19;43(3):434–44. doi: 10.1007/s11604-024-01678-1 (PMC11868203; doi:10.1007/s11604-024-01678-1)
Supplement: Supplementary file 7 — Patient characteristics in the surgery performed subgroup (NAC and Ope) [file 11604_2024_1678_MOESM7_ESM.docx]

**Supplementary Table S1** Patient characteristics in the surgery performed subgroup (NAC and Ope)

|  |  |  | Treatment |  |  |
| --- | --- | --- | --- | --- | --- |
| Variable |  | Overall | NAC | Ope | *p*-value |
| Total patients | | 67 | 49 | 18 |  |
| Sex | F | 13 (19.4) | 10 (20.4) | 3 (16.7) | 1 |
|  | M | 54 (80.6) | 39 (79.6) | 15 (83.3) |  |
| Age (years)^†^ | | 69 [47–84] | 68 [47–79] | 74 [60–84] | 0.009^*^ |
| Size (mm)^†^ |  | 60 [20–110] | 60 [30–110] | 52.5 [20–100] | 0.206 |
| i-CT | BR1 | 32 (47.8) | 23 (46.9) | 9 (50.0) | 0.02^*^ |
|  | BR2 | 13 (19.4) | 13 (26.5) | 0 (0.0) |  |
|  | BR3 | 22 (32.8) | 13 (26.5) | 9 (50.0) |  |
| cN | 0 | 5 (7.5) | 3 (6.1) | 2 (11.1) | 0.025^*^ |
|  | 1 | 17 (25.4) | 8 (16.3) | 9 (50.0) |  |
|  | 2 | 31 (46.3) | 26 (53.1) | 5 (27.8) |  |
|  | 3 | 14 (20.9) | 12 (24.5) | 2 (11.1) |  |
| Location | Cervical | 1 (1.5) | 1 (2.0) | 0 (0.0) | 0.549 |
|  | Upper | 24 (35.8) | 15 (30.6) | 9 (50.0) |  |
|  | Middle | 39 (58.2) | 30 (61.2) | 9 (50.0) |  |
|  | Lower | 3 (4.5) | 3 (6.1) | 0 (0.0) |  |
| 4DCT | R | 63 (94.0) | 45 (91.8) | 18 (100.0) | 0.567 |
|  | UR | 4 (6.0) | 4 (8.2) | 0 (0.0) |  |
| pT | 0 | 3 (4.5) | 3 (6.1) | 0 (0.0) | 0.197 |
|  | 1 | 3 (4.5) | 3 (6.1) | 0 (0.0) |  |
|  | 2 | 7 (10.4) | 6 (12.2) | 1 (5.6) |  |
|  | 3 | 50 (74.6) | 35 (71.4) | 15 (83.3) |  |
|  | 4a | 2 (3.0) | 0 (0.0) | 2 (11.1) |  |
|  | 4b | 2 (3.0) ‡ | 2 (4.1)^‡^ | 0 (0.0) |  |
| OS event | Alive | 38 (56.7) | 32 (65.3) | 6 (33.3) | 0.027^*^ |
|  | Death | 29 (43.3) | 17 (34.7) | 12 (66.7) |  |
| OS period (months)^†^ | | 18 [0–101] | 20 [0–101] | 16 [2–101] | 0.373 |

Note. Unless otherwise indicated, the data represent the number of patients.

i-CT, initial conventional CT; BR1, borderline resectable, closer to resectable; BR2, resectability not assessable; BR3, closer to unresectable; cN, clinical N factor; Location Cervical, cervical esophagus; Upper, upper thoracic esophagus; Middle, middle thoracic esophagus; Lower, lower thoracic esophagus; 4DCT, four-dimensional CT; R, resectable; UR, unresectable; NAC, neoadjuvant chemotherapy; CRT, combined chemoradiotherapy; Ope, surgery only; RT, radiotherapy; pT, pathological T factor

The Mann–Whitney U test was used for continuous data, and categorical data were compared using the chi-squared test.

^†^Data are presented as medians and ranges.

^*^Statistically significant results

^‡^Including one unresectable case at surgery
